# Statistical analysis plan for the control of blood pressure and risk attenuation-rural Bangladesh, Pakistan, Sri Lanka (COBRA-BPS) trial: a cluster randomized trial for a multicomponent intervention versus usual care in hypertensive patients

**DOI:** 10.1186/s13063-018-3022-8

**Published:** 2018-11-29

**Authors:** Mihir Gandhi, Pryseley Nkouibert Assam, Elizabeth L. Turner, Donald E. Morisky, Edwin Chan, Tazeen H. Jafar, on behalf of the COBRA-BPS Study Group

**Affiliations:** 1 Department of Biostatistics, Singapore Clinical Research Institute, #02-01, Nanos, 31 Biopolis Way, Singapore, Singapore; 2 Centre for Quantitative Medicine, Duke-NUS Medical School, Leve 6, Academia, 20 College Road, Singapore, Singapore; 3 Tampere Center for Child Health Research, University of Tampere and Tampere University Hospital, Arvo Building, Lääkärinkatu 1, Tampere, Finland; 4 Department of Biostatistics and Bioinformatics, Duke University, 2424 Erwin Road, Durham, NC USA; 5 Duke Global Health Institute, Duke University, Trent Hall, 310 Trent Drive, Durham, NC USA; 6 Department of Community Health Sciences, UCLA Fielding School of Public Health, Los Angeles, California USA; 7 Department of Epidemiology, Singapore Clinical Research Institute, #02-01, Nanos, 31 Biopolis Way, Singapore, Singapore; 8 Program in Health Services & Systems Research, Duke-NUS Medical School, 8 College Road, Singapore, Singapore

**Keywords:** Cluster randomized trial, Hypertension, Statistical analysis plan, Blood pressure

## Abstract

**Background:**

In rural south Asia, hypertension remains a significant public health issue with sub-optimal blood pressure (BP) control rates. The goal of the trial is to evaluate the effectiveness and cost-effectiveness of a multicomponent intervention (MCI) compared to usual care on lowering BP among adults with hypertension in rural south-Asian communities. This article describes the statistical analysis plan for the primary and secondary objectives related to intervention effectiveness based on clinical and patient-reported endpoints.

**Methods/Design:**

The study is a cluster randomized trial which will enroll 2550 participants aged ≥ 40 years with hypertension from rural communities in Bangladesh, Pakistan, and Sri Lanka. The unit of randomization is a cluster defined by 250–300 households. Thirty clusters, 10 from each country, are randomized in a 1:1 ratio to either MCI or usual care, stratified by country and their distance from the clinic. All participants will be assessed every six months over a two-year period after baseline with measurements of systolic and diastolic BP, antihypertensive and statin medication use, medication adherence, physical activity level, anthropometric parameters, smoking status, and dietary habits. The primary objective is to assess the effectiveness of MCI as compared with usual care in terms of mean change in systolic BP from baseline to final follow-up at two years. The primary outcome will be modelled using a generalized linear mixed-model for repeated measures based on a participant-level analysis. The model will include cluster random-effects and will use a non-independence residual correlation matrix to account for repeated measures on the same participant. Sensitivity analyses for the primary endpoint will be based on multiple imputation as well as pattern mixture model tipping point analyses. Secondary outcomes will be analyzed using the same modeling approach as for the primary outcome, with appropriate distributions within the exponential family and corresponding link functions.

**Discussion:**

The a priori statistical analysis plan will avoid reporting bias and data-driven analysis for the primary and key secondary outcomes. The results of the study will provide evidence of the benefits and risks of the MCI for BP control in rural communities in south Asian countries with low-resourced public health infrastructure.

**Trial registration:**

Clinicaltrials.gov, NCT02657746. Registered on 14 January 2016.

## Introduction

The control of blood pressure and risk attenuation-rural Bangladesh, Pakistan, Sri Lanka (COBRA-BPS) trial is a cluster randomized clinical trial to compare a multicomponent intervention (MCI) to usual care. The overall goal is to evaluate the effectiveness and cost-effectiveness of MCI in adults aged ≥ 40 years with hypertension who reside in rural communities of Bangladesh, Pakistan, and Sri Lanka. MCI comprises the following five components: (1) home health education (HHE) by government community health workers (CHWs); (2) blood pressure (BP) monitoring and stepped-up referral to a trained general practitioner (GP) using a checklist; (3) trained public and private providers in management of hypertension and using a checklist; (4) designated hypertension triage counter and hypertension care coordinators in government clinics; and (5) a financing model to compensate for additional health services and provide subsides to low-income individuals with poorly controlled hypertension. A detailed description of the COBRA-BPS trial protocol has already been published [1]. It contained a brief description of the primary effectiveness analysis. The current article describes a more detailed statistical analysis plan for the primary and secondary objectives for the intervention effectiveness based on clinical and patient-reported outcomes. At the time of writing, no post-baseline outcomes for effectiveness has been analyzed in the trial. The a priori statistical analysis plan will avoid reporting bias and data-driven analysis. This article does not include an analysis plan for other objectives such as evaluating cost-effectiveness of the intervention and patients’ experience during the course of intervention through qualitative data. These topics will be covered in separate papers.

### Randomization

**Fig. 1:**
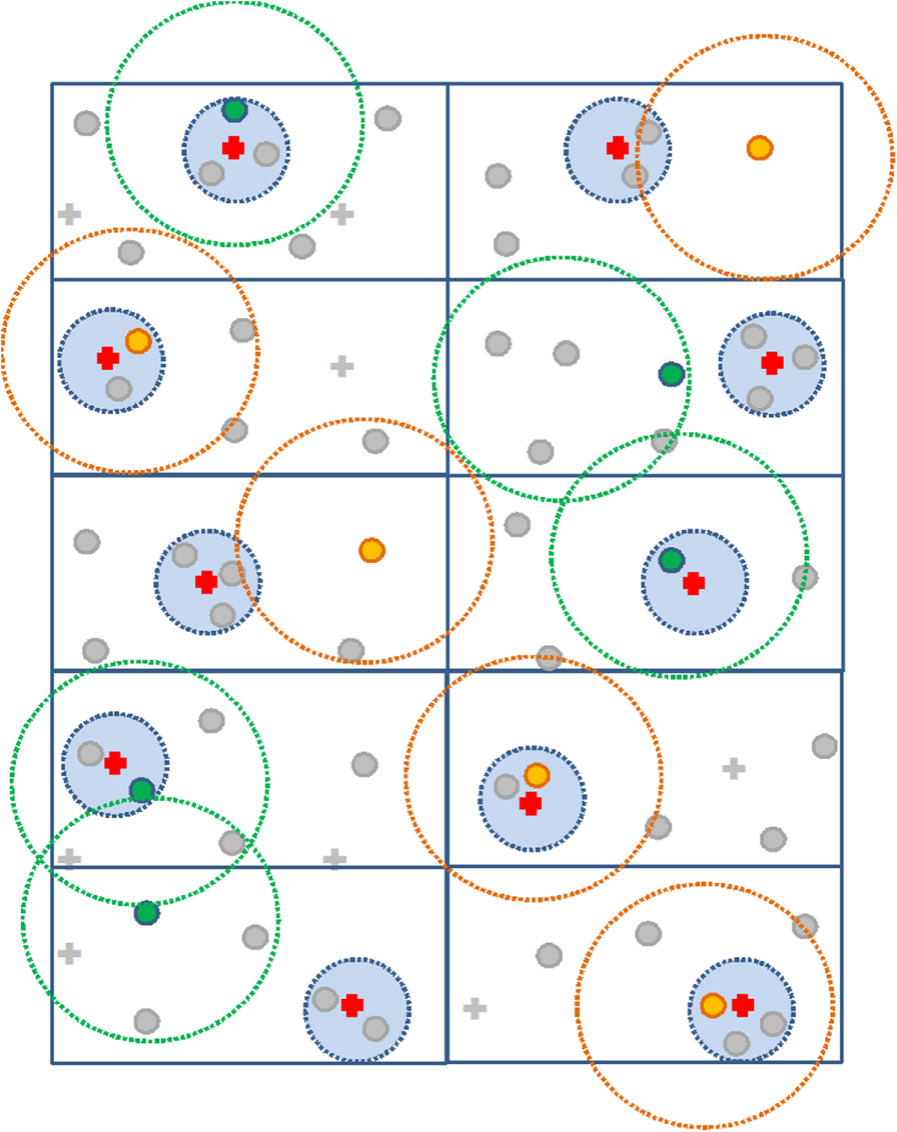
*Schematic diagram* for cluster selection criteria in the randomization  Multicomponent intervention cluster;  Usual care cluster;  Government clinic nearest to the randomized cluster;  Non-randomized cluster;  Government clinic outside 10 km radius of randomized cluster. *Rectangles* represent administrative units. *Dotted line* surrounding a cluster represents a 10-km radius to the cluster. *Dotted line* surrounding a government clinic represents a 2-km radium to the clinic. No usual care clusters are within a 10-km radius of MCI clusters. Any usual care cluster should not be nearer to a MCI government clinic than its own; similarly, any MCI cluster should not be nearer to a usual care government clinic than its own

The trial is conducted in Bangladesh, Tangail District (population 3.2 million), and Munshiganj District (population 1.4 million); Pakistan, Thatta District (population 1.5 million); and Sri Lanka, Puttalam District (population 1.6 million). The unit of randomization was a cluster defined by 250–300 households as defined by local administration according to CHW catchment area (each served by 1–2 CHWs). These clusters were grouped in geographically contiguous administrative units (AUs) as defined by the local governments (12 sub-districts in Tangail, six subdistricts in Munshignaj, 30 union councils in Thatta, 12 medical officers of the health division in Puttalam District) such that each unit is served by one government clinic. First, in the selected district of each country, 10 administrative units were deliberately sampled, and the respective government clinic was determined. Within each AU in each country, eligible clusters were identified (one cluster is defined as a village for Bangladesh, 2–5 neighboring villages for Pakistan, and two Grama Niladhari [GN] divisions for Sri Lanka). Each country measured the distance of clusters from the respective government clinic by a GPS device. In each AU, clusters were stratified into two strata according to their distance to respective government clinic: FAR and NEAR (a distance of ≤ 2 km was defined as NEAR and > 2 km as FAR). In each arm (usual care or MCI), three of the five AUs were randomly sampled to be NEAR AUs so that the remaining two were FAR. Then, one NEAR cluster from each NEAR AU and one FAR cluster from each FAR AU were randomly selected for participant recruitment. A minimum distance of 10 km between randomized clusters were ensured. Figure 1 shows a schematic diagram for cluster selection criteria. In summary, randomization was stratified by country as well as by the distance from the government clinic, and AUs (equivalently, the sampled clusters) were randomized in a 1:1 ratio to either MCI or usual care within the six strata defined by the combination of country and distance (NEAR versus FAR) using a computer-generated randomization program at Singapore Clinical Research Institute, Singapore.

### Study assessments

Study assessments are summarized in Table 1. The majority of the assessment will be performed at six-monthly home visits up to two years from the baseline. The acceptable tolerance in the six-monthly visits is ± 2 months. All assessments will be performed by independent assessors (masked to randomization status). More details of study assessment forms, questionnaires, and checklists are available in the published protocol [1].

**Table 1:** Study assessments schedule

Assessments	Study visits		
	Screening	Baseline	6-monthly (until 2 years from baseline)
Informed consent	✓		
Demographics characteristics	✓		
Systolic and diastolic blood pressures^a^	✓	✓	✓
Current blood pressure medications	✓		
Socioeconomic characteristics		✓	
Medical history		✓	
Concomitant medications		✓	✓
Family medical history		✓	
Tobacco smoking status		✓	✓
International physical activity questionnaire		✓	✓
Dietary questionnaire		✓	✓
EQ-5D-5L questionnaire		✓	✓^b^
MMAS-8 for antihypertensive medication adherence		✓	✓
MMAS-8 for statins adherence		✓	
Adiposity measures (BMI, waist circumference)		✓	✓
Laboratory tests		✓	✓^b^
Serum creatinine			
Fasting blood glucose			
Total cholesterol			
High density lipoprotein cholesterol			
Low density lipoprotein cholesterol			
Triglycerides			
Urine spot albumin			
Urine spot sodium			
Urine spot creatinine			
Adverse/serious adverse events		✓	✓
Death			✓

^a^Only measurements taken by independent assessors who will be masked to randomization will be used for the analysis

^b^To be assessed only at final follow-up visit

### Sample size

The planned sample size is 2550 participants with hypertension, corresponding to a target sample size of 85 hypertensive participants per cluster for each of the 10 clusters per country in each of the three countries. Based on findings from a previous feasibility study in rural Bangladesh, Pakistan, and Sri Lanka [2], a conservative intraclass correlation coefficient (ICC) of 0.02 was considered. Assuming 80% participant retention rate per cluster at two years after baseline (68 hypertensive participants per cluster) and a two-sided type I error rate of 5%, the trial will provide > 99% power for the overall test to detect a difference between arms in SBP reduction as small as 4 (SD 11) mm Hg [3–5]. The study will use 5 mmHg as the clinically meaningful difference between the two arms for reduction in SBP.

Pertaining to heterogeneity in the intervention effect among the three countries, we assume that < 3 mmHg difference in SBP is not clinically meaningful. Therefore, the intervention effect will be considered heterogeneous among countries if the difference in SBP reduction between any two countries is ≥ 3 mmHg (SD 11) (for example, a reduction in SBP of 3 mmHg in one country and 9 mmHg in the other two countries; or reduction in SBP is 3 mmHg in one country, 6 mmHg the in second, and 9 mmHg in the third). The planned sample size provides > 80% power to detect heterogeneity (as defined above) in intervention effects based on the following assumptions: ICC of 0.02 and type I error rate of 0.16% (based on a Bonferroni adjustment for three pairwise comparisons).

**Fig. 2:**
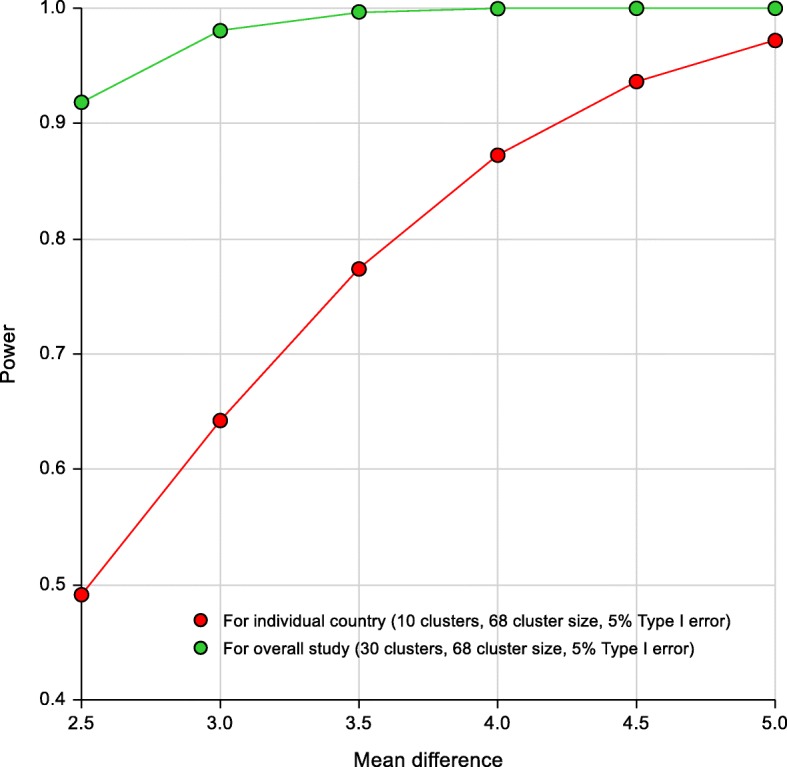
Statistical power for individual country and overall study at the planned sample size

In addition to this, the trial has > 80% power to detect a difference of 4 mmHg (SD 11) in SBP reduction between the MCI and usual care arms for each country separately, for an ICC of 0.02, a two-sided type I error rate of 5%, and 10 clusters of size 85 participants per country with 80% participant retention rate per cluster at two years post baseline (68 participants per cluster) (Fig. 2). Furthermore, the high power also ensures that the main effect is adequately powered even after adjusting for dropouts. Therefore, the study is not over-powered for heterogeneity or dropouts (missing data). Power and Sample Size (PASS) version 14 software was used for the power calculations. Based on our previous work in urban Pakistan, and expecting a somewhat lower attrition rate in rural areas, the attrition rate is likely to be < 15% at the end of two years in the overall study; therefore, our assumptions about follow-up rates are conservative [4].

### Study objectives

#### Primary effectiveness objective

To assess the effectiveness of MCI compared to usual care in terms of mean change in systolic blood pressure (SBP) from baseline to final follow-up at two years post baseline.

#### Secondary effectiveness objectives

To compare mean change from baseline, or proportion, at the final follow-up at two years post baseline between MCI and usual care arm for the following outcomes:

- BP controlled to target (SBP < 140 mmHg and diastolic blood pressure [DBP] < 90 mmHg)
- Response (SBP < 140 mmHg and diastolic BP < 90 mmHg, or ≥ 5 mmHg reduction in SBP at two years from the baseline)
- Poorly controlled BP (SBP ≥ 160 mmHg or DBP ≥ 100 mmHg)
- DBP
- Anti-hypertension and statin medications use
- Anti-hypertensive medication and statins adherence score assessed by the eight-item Morisky Medication Adherence Scale (MMAS-8)
- Physical activity score assessed by International Physical Activity Questionnaire (IPAQ)
- Cardiovascular events risk score assessed by INTERHEART “Cholesterol” modifiable risk score
- Health-related quality of life assessed by 5-Level EuroQol-5 Dimension (EQ-5D-5L) questionnaire
- Body mass index (BMI)
- Waist circumference
- Current smoking status
- Frequency of vegetables and fruits intake per week
- Salt intake assessed by urine spot sodium-to-creatinine ratio and 24-h urine sodium
- Incident diabetes
- Cholesterol level assessed by total cholesterol, high density lipoprotein cholesterol, low density lipoprotein cholesterol, and triglycerides
- Estimated glomerular filtration rate (eGFR)
- Urine albumin excretion

Table 2 lists definitions of the above outcomes at a particular visit.

**Table 2:** Outcome definitions for effectiveness objectives

Outcome	Measurement and definition
Systolic and diastolic blood pressure (SBP/DBP)	Measured using a calibrated automated device (Omron HEM-7300 Blood Pressure Monitor) with the individual in the sitting position. Three reading are taken at least 3 min apart. Mean of last two readings will be used as the final measurement.
Blood pressure controlled to target	SBP (mean of last 2 of 3 readings) is < 140 mmHg and DBP (mean of last 2 of 3 readings) is < 90 mmHg.
Response	SBP (mean of last 2 of 3 readings) is < 140 mmHg and DBP (mean of last 2 of 3 readings) is < 90 mmHg, or change in mean of last two readings from the mean of last two readings from the baseline is ≥ 5 mmHg.
Poorly controlled blood pressure	SBP (mean of last 2 of 3 readings) is ≥ 160 mmHg or DBP (mean of last 2 of 3 readings) is ≥ 100 mmHg.
Anti-hypertensive and statin medication usage	Information on any ongoing anti-hypertensive and statin medications will be classified into one of the following medication classes: Angiotensin II Receptor Blocker or Angiotensin-Converting Enzyme Inhibitor, Beta Blocker, Calcium Channel Blocker, diuretics, and statins.
Eight-item Morisky Medication Adherence Scale (MMAS-8) scores	Self-reported medical adherence is measured by the MMAS-8, separately for anti-hypertensive medication and statins [13–16]. MMAS-8 scores are calculated by summing all coded answers.
International Physical Activity Questionnaire (IPAQ) scores	Total physical activity score (MET-min/week) and activity classification (Inactive, Minimally Active, and Highly Active) are derived according to the IPAQ scoring guideline [17].
Cardiovascular events risk score	The INTERHEART “Cholesterol” modifiable risk score provides a comprehensive numeric assessment of risk factors for cardiovascular events [18]. The score is the sum of points for questions corresponding to categories of these risk factors.
Five-level EuroQol-5 Dimension (EQ-5D-5L) questionnaire index	The EQ-5D-5L is administered to assess a participant’s health status on the day of assessment. In addition, it has a visual analogue scale (VAS) measuring health on a scale of 0 (The worst health you can imagine) to 100 (The best health you can imagine) [19]. The EQ-5D-5L index summarizing health status of the participants is calculated using the EQ-5D-5L value set for England [20]. Currently, there is no value set available for Bangladesh, Pakistan, or Sri Lanka. However, if any more suitable value set becomes available before the final data analysis, it will be used. The EQ-5D-5L VAS is also considered as an additional health-related quality-of-life measure.
Body mass index	Calculated as weight (kg) divided by height^2^ (m). Height is measured using standardized Portable Stadiometer (Model SECA 213) in cm with graduation of 1 mm. Weight is measured using standardized OMRON Digital Weight Scale (Model HN-286).
Waist circumference	Measured as per the WHO STEPS protocol [21]. The measurement of waist circumference is made at the approximate midpoint between the lower margin of the last palpable rib and the top of the iliac crest.
Current smoking status	Individuals smoking tobacco on a daily basis, including cigarette, pipes, cigars, cheroots, cigarillos, and water pipe smoking sessions, are considered current smokers.
Fruits and vegetables intake	A dietary questionnaire is administered to collect information on dietary habits related to fruits and vegetables intake. At least one intake per week will be considered an indicator for each type of dietary intake.
Salt intake	Measured in terms of urine spot sodium-to-creatinine ratio and 24-h urine sodium estimation by Kawaskai formula [22]. (Chemistry analyzer [urine spot sodium]: Beckman Synchron Cx-7 by Ion Electrode; Regent [urine spot sodium]: aluminum silicate; Chemical analyzer [urine spot creatinine]: Synchron Cx-7/Delta; Regent [urine spot creatinine]: THC2)
Incident diabetes	The use of hypoglycemic agents or fasting blood glucose ≥ 126 mg/dL at any time during the two-year follow-up period for all participants without prevalent diabetes at enrollment. (Chemistry analyzer: Beckman Synchron Cx-7/Delta; Reagent: GLUCm)
Cholesterol level	Measured in terms of total cholesterol, high density lipoprotein cholesterol, low density lipoprotein cholesterol, and triglycerides. (Chemistry analyzer: Roche Hitachi 912; Reagent: Roche reagents)
Estimated glomerular filtration rate	Estimated using CKD-EPI equation [23].
Urine albumin excretion	Measured in terms of urine albumin-to-creatinine ratio defined as a ratio of spot urine albumin divided by spot urine creatinine expressed as mg/g. (Chemistry analyzer: Beckman Synchron Cx-7/Delta; Regent: Pyrogallol red plus sodium molybdate)

#### Exploratory effectiveness objectives

To compare mean change in SBP at the final follow-up at two years post baseline between MCI and usual care arms for the following sub-groups defined according to baseline characteristics:

- Participating country (Bangladesh, Pakistan, Sri Lanka)
- Cluster distance from the primary care clinic (Far, Near)
- Gender (Male, Female)
- Currently on anti-hypertensive medication (Yes, No)
- Poorly controlled BP (SBP ≥ 160 mmHg or DBP ≥ 100 mmHg)
- Socioeconomic level (Poor, Non-poor)

Additional exploratory analysis may be performed to evaluate the intervention effect for other outcomes for the abovementioned sub-groups.

#### Safety objectives

To compare the MCI and usual care arms at the final follow-up at two years post randomization for the following endpoints:

- Proportion of participants who experienced any serious adverse event (SAE)
- Proportion of participants who experienced any SAE of special interest (death [all cause], hospital admission due to coronary heart disease, heart failure, or stroke)

On event of an AE, the site investigator will decide its category and system organ class and also evaluate whether an AE is an SAE if it leads to or is classified into one or more of following categories: death; life-threatening; disability or permanent damage; hospitalization (excludes emergency room visits); prolongation of hospital stay (≥ 24 h); required intervention to prevent permanent impairment or damage; other SAEs. Table 3 lists the predefined categories and system organ class for AEs.

**Table 3:** Predefined categories and system organ classes for adverse events

Categories	System organ classes
Angioedema and anaphylactic reaction Peripheral edema Hypotension Coronary heart disease Heart failure Stroke or transient ischemic attack Headache, dizziness, or lightheadedness Flushing Cough after initiating antihypertensive Abdominal pain Muscle pain Falls and trauma Other	Systemic reactions Cardiovascular system Nervous systems Skin and appendages Respiratory system Gastrointestinal and hepatobiliary system Other

SAEs reported with an onset date before the baseline visit or after the final assessment visit at two years will not be included in the analysis.

### Potential covariates

The following baseline variables may be considered as potential covariates in the supportive analyses:

- Age (in years)
- Gender (male, female)
- Education level (no formal education, formal education)
- Marital status (single [never married, divorced, separated, widowed], not single [married])
- Socioeconomic level (poor, middle, high)
- BMI (obese/overweight [≥ 23.5 kg/m^2^ BMI], non-obese/not-overweight [< 23.5 kg/m^2^ BMI]) [6]
- Waist circumference
- Diabetes (yes, no)
- Chronic diseases (yes [heart disease, chronic kidney disease, stroke], no)
- Currently using an anti-hypertensive medication (yes, no)
- Current smoking status (yes, no)
- Physical activity score (inactive/minimally active, highly active)
- Salt intake
  - ○ Urine spot sodium-to-creatinine ratio
  - ○ 24-h urine sodium
- Cholesterol level
  - ○ Total cholesterol
  - ○ High density lipoprotein cholesterol
  - ○ Low density lipoprotein cholesterol
  - ○ triglycerides
- Kidney function
  - ○ Estimated glomerular filtration rate (eGRF)
  - ○ Urine spot albumin-to-creatinine ratio

### Potential moderators

The following variables recorded over time may be considered as potential moderators in the supportive analyses:

- BMI
- Waist circumference
- Adherence to anti-hypertensive medication
- Current smoking status
- Physical activity level

### Populations

#### Intent-to-treat (ITT) population

The ITT population consisted of all enrolled participants with a baseline visit (i.e. assessed for the primary and secondary outcomes). Participants from the MCI clusters will be included in the MCI arm even if they did not receive the MCI. Similarly, participants from the usual care clusters will be included in the usual care arm even if they are exposed to the MCI.

#### Treated population

The MCI arm includes all enrolled participants who have attended at least one interview on HHE or visited trained GPs (i.e. based on receipt of physician’s management checklist) as a part of the MCI intervention. The usual care arm includes all enrolled participants. Participants from an MCI cluster who are considered to have not been “treated” (i.e. have not attended any interview on HHE and have not visited a trained GP as a part of the MCI intervention) will be analyzed with the usual care arm.

#### Per-protocol population

The per-protocol population consists of all enrolled participants who do not have any significant protocol deviations (described below).

Significant protocol violation/deviations including those which could have an impact on the primary effectiveness measures, those which present a safety risk to the participants, and/or those that are of ethical concern will be identified during a blinded data review before database lock.

General significant protocol violation/deviation criteria are listed below:

- Eligibility deviations (included in the study despite meeting following criteria)
  1. Wrong hypertensive diagnosis: neither has persistently uncontrolled BP (SBP ≥ 140 mmHg or DBP ≥ 90 mmHg) nor on anti-hypertensive medications.
  2. Under age: aged < 40 years.
- On-study deviations (continued the study despite meeting following criteria)
  1. Pregnancy
  2. Any major medical systemic illness which precludes continuation
  3. Error in intervention assignment: participants treated with intervention arm different from the assigned cluster’s intervention arm.
  4. Withdraw consent or lost to follow-up

The above list of significant protocol deviation criteria may be extended as appropriate.

All effectiveness analyses will be performed using the ITT population. However, considering the nature of the study (community-based cluster randomized trial with data collection in rural parts of three developing countries), there is a chance that a small proportion of participants may not provide any follow-up data and hence they will not contribute to the evaluation of intervention effectiveness. If this proportion of participants is sizable, participant disposition, and demographic and baseline participant characteristics will be analyzed using the ITT population, as well as excluding participants who have not contributed in the effectiveness analyses. The ITT and per-protocol populations may be used for additional supportive analysis of effectiveness endpoints. Study intervention exposure and safety analyses will be performed using the treated population. The ITT population may also be used for supportive safety analyses.

### Statistical analyses

#### General methods and data handling rules

All observed data will be included in the ITT population, with the exception of data collected outside the acceptable assessment window (± 2 months) for each time point and participants with no follow-up data. The frequency distribution of the effectiveness outcomes will be reviewed, e.g. using boxplots and histograms. Continuous variables with excessive skewness and/or kurtosis will be analyzed using appropriate methods for asymmetric data or considered for transformation. All *p* values will be two-sided. A *p* value < 0.05 for the primary analysis will be considered statistically significant, in line with the prespecified level used in the sample size calculation. All confidence intervals (CI) will be at the 95% level. All statistical analyses will be carried out using SAS software (SAS Institute, NC, USA). After the statistical plan has been written and signed off, and after the database for the final analysis has been locked, the individual cluster’s assigned intervention will be made known to the study team.

#### Trial profile

The number of participants enrolled into the study at screening, reasons for screening failure, number of participants enrolled, number of participants who completed the baseline and follow-up visits, mean and SD of cluster size at each visit will be summarized by intervention arm using the CONSORT flow chart [7]. The distribution of baseline characteristics will be summarized by: (1) intervention arm; and (2) country and intervention arm, with descriptive statistics for the ITT population.

#### Intervention exposure

MCI exposure is evaluated using the HHE session delivery rate, physician referral rate, and physician’s evaluation rate. Table 4 defines these fidelity measures. The fidelity measures are estimated along with corresponding 95% CI for country for the MCI arm based on the treated population. Exposure and adherence to anti-hypertensive medications is summarized as secondary outcomes.

**Table 4:** Intervention fidelity measures

Fidelity measure	Definition
Home health education (HHE) session delivery rate	Calculated as the total number of three-monthly HHE sessions delivered at the household level using the *Community Health Workers Monitoring and Home Health Education Checklist* divided by the total number of planned HHE sessions until the study discontinuation/completion, multiplied by 100.
Physician referral rate	Calculated as the total number of times they are referred to trained physicians by CHWs using *the General Practitioner Referral Checklist* divided by the total number of times participants identified with having poorly controlled BP (SBP ≥ 160 mmHg or DBP ≥ 100 mmHg) during study visits until the study discontinuation/completion, multiplied by 100.
Physician’s evaluation rate	Calculated as the total number of times they are evaluated by trained physicians using *the General Practitioner Management Checklist* divided by the total number of times participants identified with having poorly controlled BP during study visits until the study discontinuation/completion, multiplied by 100.

#### Primary effectiveness analysis

This primary analysis will be performed on the ITT population. Change in SBP at two years from baseline is the primary outcome. The four six-monthly change-from-baseline measurements (six months, 12 months, 18 months, and 24 months) from all participants will be modelled simultaneously using a likelihood-based generalized linear mixed-model for repeated measures (MMRM) based on a participant-level analysis, incorporating a cluster random-effect, using Gaussian distribution and identity link function [8, 9]. Appropriate distributions within the exponential family and corresponding link functions will be employed for the primary outcome in case of non-normality. An unstructured matrix will be used to model the residual variance-covariance structure within participant. If this model fails to converge, heterogeneous toeplitz, heterogeneous autoregressive of order one, autoregressive of order one, and compound symmetry structures will be considered in the specified order to model the correlation between time points from the same participant. MMRM accounts for missing data and is valid under the missing at random (MAR) assumption.

All primary analysis models will include fixed effects for baseline SBP, country, indicator for distance from clinic (far or near), age, gender, intervention arm, visit number, and the intervention arm-by-visit number interaction. The primary outcome of interest at two years from baseline will be estimated with corresponding 95% CIs using the appropriate contrast at the final visit. We will employ restricted/residual maximum likelihood with the between-within approximation for degree of freedom estimation [10].

#### Supportive effectiveness analyses

A MMRM model will be performed for SBP similar to the primary analysis mentioned above including interactions for country, intervention group, and time to determine whether the effects differ by country. If the interaction effect is found to be clinically meaningful, a MMRM model similar to the primary analysis model will be performed separately for each country. The country-specific analyses will use Bonferroni corrected *p* values and CIs for evaluating intervention effectiveness. Further analysis will be performed similar to the primary analysis with each potential confounder at a time as fixed effect (separate model for each confounder) as well as all (or selected) potential confounders at the same time as fixed effects (single model including multiple confounders). The final list of confounders will be decided considering statistical significance (*p* < 0.1), effect size, and clinical importance. A MMRM model will be performed for SBP similar to the primary analysis mentioned above, including each moderator at a time as fixed effect as well as all moderators at the same time as fixed effects using the ITT population. Further supportive analysis may be performed using the ITT and per-protocol populations.

#### Secondary effectiveness analyses

Secondary effectiveness outcomes will be analyzed using a similar strategy applied to the primary endpoint. That is, using a MMRM with an unstructured variance-covariance matrix. Appropriate distributions within the exponential family and corresponding link functions will be employed for each outcome. Analysis of incident diabetes (for those without prevalent diabetes at baseline), salt intake, and cholesterol level will be based on only two time-points (baseline and two years) using similar MMRM models. All secondary analyses will be performed on the ITT population. Supportive analyses may be performed using the treated and per-protocol populations. Additional supportive analyses may be performed to evaluated intervention effect after adjusting for potential confounders.

#### Exploratory effectiveness analyses

A MMRM model will be performed for SBP similar to the primary analysis mentioned above including a fixed effect term for currently anti-hypertensive medication (yes/no) at baseline and its interactions with intervention group and time to determine whether the intervention effect differs by anti-hypertensive medication use status at baseline. Similar analysis will be performed for cluster distance from the primary care clinic, gender, poorly controlled BP status, and socioeconomic status at baseline. If the interaction effect is found to be clinically meaningful, a MMRM model similar to the primary analysis model will be performed separately for each sub-group. These analyses will be performed on the ITT population.

#### Handling of missing data in the effectiveness analyses

The primary analysis is planned with no imputation for missing data. The primary analysis, based on a likelihood-based MMRM, is valid under the MAR assumption [11]. The MAR assumption means that missingness is independent of the unobserved outcome values after accounting for the appropriate observed data and covariates in the model.

In order to evaluate the robustness of the findings to the MAR assumption, sensitivity analyses will be performed under varying assumptions for data considered likely to be missing under MAR, as well as missing not at random (MNAR). MNAR means that missingness depends on the unobserved values and cannot be predicted solely based on the participant’s observed data. Several types of statistical models have been proposed to analyze clinical study data under such assumptions. We will use two possible approaches to evaluate the impact of missingness.

The first approach that will be implemented for this study is the use of multiple imputations and MMRM for the primary outcome. Using MI data for change in SBP post baseline (six months, 12 months, 18 months, and 24 months from baseline measurements) from all participants will be modelled simultaneously using MMRM model same as the primary analysis.

It is expected that the majority of missing data will be caused by participants discontinuing from the study prematurely. The resulting missing data will have a monotone pattern, meaning that once a participant has missing data for a visit, data will be missing for all subsequent visits. It is also expected that a small amount of non-monotone missing data (when participants skip intermediate visits but return for evaluations at subsequent visits) will be present. The intermittent missing data will be imputed using the Monte Carlo Markov Chain (MCMC) method for multiple imputation before the imputation of the monotone missing data [8].

The second approach is the use of pattern mixture models (PMMs) and multiple imputations, which will be implemented if the amount of missingness on the primary outcome is > 25% or the difference in percentage of missing data between the intervention arms is ≥ 15% [8]. PMMs have the advantages of allowing transparent and clinically interpretable formulations of the assumptions regarding unobserved data [11, 12]. PMMs with delta (δ) adjustments will be used and imputations will be based on an MNAR clinical assumption that participants from the MCI arm who discontinue at a given time point would have, on average, their unobserved efficacy score worse by some amount, δ, compared with the observed efficacy score of participants who continue to the next assessed time point. For purposes of the sensitivity analyses, participants who discontinue from the usual care arm are treated as if they would have exhibited the same evolution of the disease and same benefit from intervention with usual care as participants that stayed on the study. Delta values will be based on the estimated intervention effect taken from the primary analysis in the ITT population where values will vary from 0 to the estimated intervention difference in increments of 0.5 mmHg, so that one can assess at which point the study conclusions change from favorable to unfavorable, that is, so that one can find a tipping point. These will be based on 10 imputations δ-value. The magnitude of the tipping point will then be interpreted clinically and the robustness of the study conclusions to missingness evaluated.

#### Safety analysis

All on-study SAEs reported on SAE reporting forms will be summarized by intervention arm, using the treated population. The percentage and frequency of participants who ever reported each type of SAE and SAE of special interest along with system organ class will be tabulated. A similar safety analysis may be performed on the ITT population. All deaths, together with reasons, will be summarized using counts by intervention arm based on the ITT population.

#### Interim analyses

Planned interim safety analyses are to occur at every six months from the start of the study. The interim analyses will summarize participant baseline characteristics and on-study safety data by randomized group, as well as pooled over all randomized groups, for the treated population. The by randomized group analysis is reviewed by an independent data safety and monitoring board. The study team, except statisticians involved in performing the interim analyses, is blinded to these results. The pooled analysis (combined of randomized groups) is presented to the study team.

## Discussion

The COBRA-BPS trial is a pragmatic, multi-country, cluster randomized, controlled trial of a MCI with potential to implement it on a wide scale nationally in the participating countries and beyond. The study aims to evaluate the benefits of the MCI and monitor potential safety concerns for BP control in the rural communities in south-Asian countries with low-resourced public health infrastructures.

The cluster randomized study design is a pragmatic study to mimic how the proposed intervention can be rollout primarily using the existing infrastructure in countries with different types of healthcare systems and availability of resources. Therefore, it helps to evaluate not just the effectiveness of the intervention in the real-life setting but also its feasibility in implementation with an estimate of required resources, time and costs.

The study is planned to collect comprehensive data covering the impact of the intervention on change in participants’ BP control, anthropometry, life-style, diet, medication, and health-related quality of life as well as costs associated with medications and other medical events. This holistic approach will help determine the direct and indirect impact of the intervention on overall health.

The primary statistical analysis will be performed using the ITT principle. It will provide an estimate of effectiveness of the intervention in presence of varying level of adherence to the intervention and other protocol deviations. We have also planned to perform the analysis based on the per-protocol population which will provide an estimate of in an ideal and more controlled setting. Further sensitivity analyses will help to explore the impact of the intervention under varying statistical assumptions, controlling potential confounders and subgroups of special interest.

As the MCI involves components such as HHE by government CHWs, BP monitoring, and stepped-up referral by GPs, we expect that there will be some over- and under-enthusiastic CHWs; also, variability in adherence to the clinical guideline by GPs means the effectiveness of the intervention may be impacted. However, as the intervention is designed to roll out for a population-wide program, variation in performance of individual CHWs and GPs are of limited interest. Therefore, multilevel modeling incorporating such micro-level effects is not considered as the primary analytic approach. However, the main analysis is designed to incorporate cluster and county level effects in the analysis.

In summary, we have presented the statistical analysis plan to evaluate the real-world impact of the MCI before any post-baseline outcomes for effectiveness have been analyzed. The a priori statistical analysis plan will avoid reporting bias and data-driven analysis for the primary and key secondary outcomes.

## Abbreviations

AE: Adverse event
AU: Administrative unit
BMI: Body mass index
BP: Blood pressure
CHW: Community health worker
COBRA-BPS: The control of blood pressure and risk attenuation-rural Bangladesh, Pakistan, Sri Lanka
DBP: Diastolic blood pressure
eGFR: Estimated glomerular filtration rate
EQ-5D-5L: EuroQol-5 Dimension-5 Level questionnaire
GP: General practitioner
HHE: Home health education
ICC: Intraclass correlation coefficient
IPAQ: International physical activity questionnaire
ITT: Intent-to-treat
MAR: Missing at random
MCI: Multicomponent intervention
MCMC: Monte Carlo Markov Chain
MMAS-8: Morisky medication adherence scale
MMRM: Mixed-model for repeated measures
MNAR: Missing not at random
PASS: Power and sample size
PMM: Pattern mixture model
SAE: Serious adverse event
SBP: Systolic blood pressure
SD: Standard deviation
SOC: System organ class
VAS: Visual analogue scale
